# A potential small-molecule synthetic antilymphangiogenic agent norcantharidin inhibits tumor growth and lymphangiogenesis of human colonic adenocarcinomas through blocking VEGF-A,-C,-D/VEGFR-2,-3 “multi-points priming” mechanisms *in vitro* and *in vivo*

**DOI:** 10.1186/s12885-015-1521-5

**Published:** 2015-07-19

**Authors:** Xin-Ping Li, Wei Jing, Jian-Jun Sun, Zhong-Yan Liu, Jing-Tao Zhang, Wei Sun, Wei Zhu, Yue-Zu Fan

**Affiliations:** 1 Department of Surgery, Tongji Hospital, Tongji University School of Medicine, Tongji University, Shanghai, 200065 People’s Republic of China; 2 Department of Surgery, Shanghai Tenth People’s Hospital, Tongji University School of Medicine, Shanghai, 200072 People’s Republic of China; 3 Department of Surgery, Dahui Hospital, Postgraduate on-the-job in 2011 Grade, Tongji University School of Medicine, Shanghai, 200237 People’s Republic of China

**Keywords:** **C**olonic neoplasm, Norcantharidin, Tumor growth, Lymphangiogenesis, Antilymphangiogenic therapy

## Abstract

**Background:**

Tumor lymphangiogenesis plays an important role in promoting growth and metastasis of tumors, but no antilymphangiogenic agent is used clinically. Based on the effect of norcantharidin (NCTD) on lymphangiogenesis of human lymphatic endothelial cells (LECs), we firstly investigated the antilymphangiogenic activity of NCTD as a tumor lymphangiogenic inhibitor for human colonic adenocarcinomas (HCACs).

**Methods:**

*In vivo* and *in vitro* experiments to determine the effects of NCTD on tumor growth and lymphangiogenesis of the *in-situ* colonic xenografts in nude mice, and lymphatic tube formation of the three-dimensional (3-D) of the co-culture system of HCAC HT-29 cells and LECs were done. Proliferation, apoptosis, migration, invasion, Ki-67, Bcl-2 and cell cycle of LECs and the co-culture system *in vitro* were respectively determined. Streparidin-peroxidase staining, SABC, western blotting and RT-PCR were respectively used to examine the expression of LYVE-1, D2-40, CK20 (including their LMVD), and VEGF-A, VEGF-C, VEGF-D, VEGFR-2 and VEGFR-3 *in vitro* and *in vivo*.

**Results:**

NCTD inhibited tumor growth and lymphangiogenesis of the *in-situ* colonic xenografts *in vivo*, and these observations were confirmed by facts that lymphatic tube formation, proliferation, apoptosis, migration, invasion, S-phase cell cycle, and Ki-67 and Bcl-2 expression *in vitro*, and LYVE-1, D2-40, CK20 expression and their LMVD *in vitro* and *in vivo* were inhibited and affected. Furthermore, the expression of VEGF-A, VEGF-C, VEGF-D, VEGFR-2 and VEGFR-3 at protein/mRNA levels in the process of lymphatic tube formation *in vitro* and tumor lymphangiogenesis *in vivo* was downregulated; NCTD in combination with mF4-31C1 or Sorafenib enhanced these effects.

**Conclusions:**

NCTD inhibits tumor growth and lymphangiogenesis of HCACs through “multi-points priming” mechanisms i.e. affecting related malignant phenotypes, inhibiting Ki-67 and Bcl-2 expression, inducing S-phase cell cycle arrest, and directly or indirectly downregulating VEGF-A,-C,-D/VEGFR-2,-3 signaling pathways. The present finding strongly suggests that NCTD could serve as a potential antilymphangiogenic agent for tumor lymphangiogenesis and is of importance to explore NCTD is used for antitumor metastatic comprehensive therapy for HCACs.

## Background

Metastatic spread of tumor cells is the most lethal aspect of cancer and often occurs *via* the lymphatic vessels, whereas lymphangiogenesis refers to the formation of lymphatic vessels from preexisting lymphatic vessels, which plays an important role in promoting growth and metastatic spread of tumor cells [25]. The tumor-associated lymphatic vessel, also referred to as tumor lymphangiogenesis, is the growth of newly formed lymphatic vessels in cancer; this process with multiple steps is similar to the well-known mechanism of angiogenesis including endothelial cell proliferation, migration, rearrangement and tube formation, along with degradation, reconstruction and production of extracellular matrix; thus tumor lymphangiogenesis acts as a conduit by which disseminating tumor cells access regional lymph nodes and form metastases [25, 31, 43]. VEGF family, which consists of VEGF-A, VEGF-B, VEGF-C, VEGF-D and placental growth factor (PGF), contributes to vasculogenesis composed of neoangiogenesis and lymphangiogenesis. Roskoski R Jr. reviewed the interaction of several ligands and VEGF family of receptors, which consists of three protein-tyrosine kinases (VEGFR-1,-2 and-3) and two non-protein kinase co-receptors (neuropilin-1,-2) [39]. Extensive studies have showed that tumor- or stromal-secreted cytokines such as VEGF-C and VEGF-D, and their cognate receptor tyrosine kinase VEGFR-3 located on LECs are critical regulators of lymphangiogenesis, these molecules advance or regulate proliferation, migration, metastasis and survival of LECs, growth of new lymphatic capillaries and lymphatic tube formation in tumorigenesis, thus promote metastatic spread of tumor cells to lymph nodes [20, 28]. Therefore, inhibition of tumor lymphangiogenesis or its VEGF-C,-D/VEGFR-3 signaling pathways may be potential therapies for primary tumors and metastasis *via* the lymphatics. VEGF-A and VEGF-B, and their cognate receptor tyrosine w-kinase VEGFR-1 and VEGFR-2 are regarded as most important regulators of angiogenesis and key targets of antiangiogenesis [20, 48]. However, there is a crosstalk between angiogenesis and lymphangiogenesis in tumor progression [41]. Nagy et al. have demonstrated that in addition to angiogenesis, VEGF-A also induces proliferation of lymphatic endothelium, resulting in the formation of greatly enlarged and poorly functioning lymphatic channels, and abnormal lymphangiogenesis; these findings raise the possibility that abnormal lymphangiogenesis may also be expected in other circumstances such as malignant tumors characterized by VEGF-A overexpression [32]. Thus in the design of anti-lymphangiogenesis, in addition to the VEGF-C,-D/ VEGFR-3 signaling pathways, the VEGF-A or -B/VEGFR-2 signaling pathways should be considered as potential therapy targets for primary tumors and metastasis.

A growing body of evidence has indicated that traditional Chinese medicines contain anticancer ingredient. NCTD (7-oxabicyclo [2.21] heptane-2, 3-dicarboxylic anhydride) is a demethylated derivative of cantharidin with antitumor properties, which is an active ingredient of the traditional Chinese medicine Mylabris, and is a small-molecule, low-cytotoxic compound synthesized from furan and maleic anhydride *via* the Diels Alder reaction [15, 49]. It has been reported that NCTD not only effectively inhibits the proliferation and growth of a variety of human tumor cells *in vitro* and *in vivo*, but also is used selectively in clinic to treat hepatic, gastric, colorectal and ovarian carcinomas and leucopenia in China because of its effective anticancer activity, fewer side effects and leukocytosis [3, 9, 12, 19, 60]. Some experiments have also showed that NCTD plays an important role in antiangiogenesis and anti-vasculogenic mimicry for some carcinomas [4, 51, 61–63]. However, the antitumor lymphangiogenic role of NCTD in tumor lymphangiogenesis and lymphatic metastasis, and the related molecule mechanism are not still elucidated, and so far no similar studies have been published. Recently, we reported the inhibitory effect of NCTD on lymphatic tube formation, i.e. lymphangiogenesis of human LECs and the underlying mechanisms *in vitro* [23]. Here, we further investigated the effects of NCTD on lymphatic tube formation of the co-culture system consisting of HCAC HT-29 cells and LECs i.e. primary human dermal lymphatic endothelial cells (HDLECs) *in vitro*, tumor growth and lymphangiogenesis of the *in-situ* colonic xenografts in nude mice *in vivo*, and the related signaling pathways such as VEGF-C, −D/VEGFR-3 and possible crosstalk pathway VEGF-A/VEGFR-2 *in vitro* and *in vivo*, so as to explore that it is whether served as a target inhibitor for tumor lymphangiogenesis and lymphatic metastasis, and a potential small-molecule synthetic antilymphangiogenic agent for HCACs.

## Methods

### Cell lines and cultures

Human colonic adenocarcinoma HT-29 cell lines were provided by the Institute of Cell and Biochemistry, Chinese Academy of Sciences (Shanghai, China), and grown in RPMI-1640 medium supplemented with 10 % fetal bovine serum (FBS; Gibco, USA) in an incubator (Forma Scientific, USA) at 37 °C under a mixture of 95 % air and 5 % CO_2_.

Human lymphatic endothelial cells were primary HDLECs purchased from ScienCell Research Laboratories, USA. Cells were identified by immunefluorescent cytochemical technique *via* CD31, Podoplanin and LYVE-1, and grown in endothelial cell growth medium (ECGM) with endothelial cell growth factor (ScienCell Research Laboratories) in an incubator (Forma Scientific) with 5 % CO_2_ at 37 °C as described previously [23], then were used in the experiments at fifth generation of the cells.

### In-situ colonic xenograft assay and survival analysis *in vivo*

This study was carried out in strict accordance with the official of Chinese Guide for the Care and Use of Laboratory Animals and the ARRIVE (Animal Research: Reporting of *In Vivo* Experiments) guideline [18] in order to investigate the inhibitory effect of NCTD on HCACs by *in-situ* xenograft assay and survival analysis *in vivo.* The protocol was approved by the Ethics Committee of Animal Experiments of Tongji Hospital, Tongji University School of Medicine and the Science and Technology Commission of Shanghai Municipality (Permit Number: SYXK 2012–0031).

Balb/c nu/nu mice (male mice, 5 ~ 6-week old, about 20 g) from the Shanghai Laboratory Animal Center, China) were housed in specific pathogen free (SPF) condition. *In-situ* colonic xenograft and the xenograft lymphangiogenic model of HT-29 cell lines in nude mice were established as described previously [47]. Xenograft mice were randomly divided into a control group, receiving intraperitoneal (i.p.) injections of 0.2 ml sterile saline and administration through gastric tube of 0.1 ml sterile saline once two days for 6 weeks, a NCTD group, a Sorafenib group and a NCTD + Sorafenib group (20 mice per group), in which each mouse respectively received i.p. injection of NCTD (28 mg/kg, a dose of 1/5 LD_50_ [61]; No. GYZZ- H20064531, Injection solution, 5 mg/ml, Jiangsu Yew Pharmaceutical Co., Ltd, Wuxi, China) given in 0.2 ml sterile saline and administration through gastric tube of 0.1 ml sterile saline, i.p. injection of 0.2 ml sterile saline and administration through gastric tube of Sorafenib (40 mg/kg; Sorafenib Tosylate Tablets, 0.2 g/tablet, Bayer HealthCare AG, Germany) given in 0.1 ml sterile saline, or simultaneously i.p. injections of 28 mg/kg NCTD and administration through gastric tube of 40 mg/kg Sorafenib, once two days for 6 weeks in all. The xenograft size was measured with calipers two times each week. Of xenograft mice in each group, one half were sacrificed under anesthesia at 8 weeks after agent administration, tumor growth including tumor volume, tumor growth curve and tumor inhibitory rate were evaluated, and tumor morphology such as hematoxylin and eosin (H&E) staining, immunohistochemical staining and microstructures were observed under an inverted light microscope (Olympus IX70, Japan) and a TEM (JEM-1230, JEOL, Japan), respectively, as described previously [23, 47, 61]; other half of xenograft mice continued to be housed in SPF condition, and their survivals were evaluated. Mice outcome was followed from the date of drug administration to the date of death. The median follow-up period for mice was 16 (range, 3–30) weeks.

### Lymphangiogenic and lymphatic micrometastic assays of the in-situ colonic xenografts *in vivo*

In the experiment, tumor lymphangiogenesis and lymphatic micrometastasis of the *in-situ* colonic xenografts *in vivo* including lymphatic specific marker LYVE-1, D2-40 and lymphatic micrometastic marker CK20 at protein and mRNA levels, and LMVD were determined by using SABC immunohistochemical staining, western blotting and fluorescent quantitative RT-PCR as described previously [47]. As shown in Table 1, PCR amplifications were performed with LYVE-1, D2-40 gene-specific primers designed and synthesized by Invitrogen (USA).

**Table 1:** Lymphangiogenic signaling-related and lymphatic specific markers

Cells	Genes	PCR primers (forward-reverse)
HCACCs and the co-culture system *in vitro*	VEGF-A	5′-*CAC CGC CTC GGC TTG TCA CAT-3*′
		5′-*CTG CTG TCT TGG GTG CAT CTG*-3′
	VEGF-C	5′*-ACC TGC CCC ACC AAT TAC A*-3′
		5′-*GCC TCT TGT AAA GAC TGG TT*-3′
	VEGF-D	5′-*GCT GTT GCA ATG AAG AGA CG*-3′
		5′-*TCT TCT GTT CCA GCA AGT GC*-3′
	VEGFR-2	5′-*CAC ACA GAG ATG ATT ACT ACA CT*G-3′
		5′-*CCA TCT TGA GCA TCA GAT CC TC*-3′
	VEGFR-3	5′-*AAG TAC ATC AAG GCA CGC ATC GAG*-3′
		5′-*GGC TTG TTG ATG AAT GGC TGC TCA*-3
	GAPDH	5′-*ACA GAG CCT CGC CTT TGC C*-3′
		5′-*CAT GTC GTC CCA GTT GGT G*-3′
*In-situ* xenograft cells *in vivo*	VEGF-A	5′-*CTG CTC GCC GCT GCG CTG*-3′
		5′-*GTG CTG GTG TTC ATG CAC TGC AG-*3′
	VEGF-C	5′-*GCC ACG GCT TATG CAA GCA AAG AT*-3′
		5′-*AGT TGA GGT TGG CCT GTT CTC TGT*-3′
	VEGF-D	5′-*CGA TGT GGT GGC TGT TGC AAT GAA*-3′
		5′-*GCT GTT GGC AAG CAC TTA CAA CCT*-3′
	VEGFR-2	5′-*CGG AGT CAA CGG ATT TGG TCG TAT*-3′
		5′-*AGC CTT CTC CAT GGT GGT GAA GAC*-3′
	VEGFR-3	5′-*GAC AGC TAC AAG TAC GAG CAT CTG*-3′
		5′-*CGT TCT TGC AGT CGA GCA GAA*-3′
	CK-20	5′-*CAG ACA CAC GGT GAA CTAT GG*-3′
		5′-*GAT CAG CTT CCA CTG TTA GAC G*-3′
	LYVE-1	5′-*TGC AGA ATT ATG GGG ATC AC*-3′
		5′-*GGC TGT TTC AAC TTG GTC CT*-3′
	D2-40	5′-*GGT GCC GAA GAT GAT GTG*-3′
		5′-*CGA TGC GAA TGC CTG TT*A-3
	GAPDH	5′-*GCA CCA CCA ACT GCT T*A-3′
		5′-*AGT AGA GGC AGG GAT GAT*-3′

### Lymphatic tube formation assay and lymphatic marker determination of HDLECs and co-culture *in vitro*

In the experiment, the lymphatic capillary-like structures formed from the 3-D culture of HDLECs and the co-culture system, and the expression of LYVE-1 and D2-40 from these cultures and co-cultures *in vitro* were observed and determined. 24-well plates by using Transwell chambers with polycarbonate filters (pore size 0.4 μm, diameter 6.5 mm) were used. HT-29 cells (1 × 10^5^ cells/ml) were added to or not added to the upper compartment of the chamber; HDLECs (5 × 10^4^ cells/ml) were added to the lower compartment of the chamber in which bottom prior to the laying of Matrigel matrix (Becton Dickinson, USA) (200 μl/per chamber). The medium was changed every 2 days. After 1 week, cells were untreated (control group, equal ECGM solution) or treated with 2.5 μg/ml NCTD (NCTD group; about 1/3 IC_50_ for HDLECs [37]), 100 μl mF4-31C1 (Epitomics, USA; mF4-31C1 group) and NCTD + mF4-31C1 (NCTD + mF4-31C1 group) (6 chambers per group), respectively, in fresh culture medium in an incubator (Forma Scientific) with 5 % CO_2_ at 37 °C for 2 ~ 4 days. The effects on lymphatic tube formation including the capillary-like structures, the total number of cell clusters and branching of tube formation (i.e., capillary-tube number) of each group were observed using an inverted phase-contrast light microscope (Olympus IX70) as described previously [23]. At the same time, the expression of LYVE-1 and D2-40 from the 3-D culture or co-culture was determined using western blotting as described previously [23, 47]. These experiments were performed in triplicate.

### Proliferation and proliferating marker Ki-67 assays *in vitro*

Methyltiazolyl tetrazolium (MTT; Sigma, USA)-based colorimetric assay was used to evaluate the inhibitory effect of NCTD on proliferation of HT-29 cells, HDLECs and the co-culture system in *vitro*. The cultures were divided into a NCTD group and a control group. HT-29 cells (1 × 10^5^ cells/ml, 100 μl/well) were cultured in 24-well plates in RPMI-1640 medium (100 μl/well), and HDLECs (5 × 10^4^ cells/ml, 100 μl/well) were cultured in fibronectin-coated 24-well plates in ECGM medium (100 μl/well). Proliferation assay for the co-culture system, 24-well plates by using Transwell chambers with polycarbonate filters (pore size 0.4 μm, diameter 6.5 mm) were used; HT-29 cells (1 × 10^5^ cells/ml) were added to the upper compartment of the chamber, HDLECs (5 × 10^4^ cells/ml) to the lower compartment of the chamber (200 μl/per chamber). Cells then were untreated (control group, equal RPMI-1640 or ECGM solution) or treated with various concentrations (1.25 ~ 100 μg/ml; 6 wells per concentration) of NCTD (NCTD group) in fresh culture medium at 37 °C in 5 % CO_2_ for 24 h. The optical densities (*A* value) at 490 nm were measured with an enzyme-linked immunosorbent assay reader (Elx800UV, Bio-Tek, USA). The *A*490 value of the experimental groups was divided by the *A*490 value of untreated controls and presented as a percentage of the cells. The inhibitory percent of NCTD on the cells (%) = (1- *A*490 value in the experimental group/*A*490 value of control group) × 100 %. Three separate experiments were performed. The concentration of drug giving 50 % growth inhibition (IC_50_) was calculated from the formula IC_50_ = lg^−1^{Xm-I [P-(3-Pm-Pn)/4]}.

In order to further observe the inhibitory effect of NCTD on proliferation of HDLECs and the co-culture system, proliferation marker Ki-67 of above LYVE-1 or D2-40-positive HDLECs and co-culture system in *vitro* were determined by SABC immunocytochemical staining as described previously [4]. Cells plated on slides were untreated (control group, equal RPMI-1640 or ECGM solution) or treated with an 1/3 IC_50_ dose of NCTD (NCTD group), and primary antibody of Ki-67 (mouse monoclonal antibody, 1:100, Antibody Diagnostica Co., USA) was added, then biotinylated secondary antibody (goat anti-rabbit IgG, 1:100), SABC reagents and DAB solution (all from Boster Co., China). For negative control, the slides were treated with PBS in place of primary antibody. Then, cells were rinsed in distilled water, dehydrated through alcohol and xylene and mounted on a coverslip using a permanent mount medium for analysis by a microspectrophotometer (Leitz Dmrbe, Leica). Ten sample slides in each group were chosen for analysis. More than 10 visual fields were observed or more than 500 cells were counted per slide. The positive index of Ki-67 represented expression of Ki-67 protein. The stain integral of Ki-67 protein was counted according to the positive number and the intensity of staining of the cells.

### Apoptosis and apoptotic gene Bcl-2 assays *in vitro*

Immunofluorescent dye, FCM and TEM were used in this assay as described previously [23]. Cell culture and experiment were performed according to above proliferation assay. For immunofluorescent dye, cells were fixed, washed and stained with 0.5 ml fluorescence agent Hoechst 33258 (Sigma) and CY3 NHS ester (Lumiprobe, USA), then observed and counted for cell apoptotic percent of each group under a fluorescence microscope (Nikon Eclipse TE2000-U, Japan) as described previously [37]. For FCM, cells (5 × 10^5^ cells/ml) suspended in 500 μl binding buffer were used for DNA stain with 5 μl Annexin V-FITL and propidium iodine (PI, Sigma); then, DNA value, cell cycle and apoptotic rate of each group were determined by a cell apoptotic detection kit (BioDev, China) and a fluorescent activated cell sorter (420 type FCM, Becton-Dickinson, USA) as described previously [9, 12, 23]. Cells were observed under an inverted microscope (Olympus IX70) and a TEM (JEM-1230, JEOL) as described previously [23].

In addition, in order to further observe the inducing effect of NCTD on apoptosis of HDLECs and the co-culture system, anti-apoptotic gene Bcl-2 of HDLECs and co-culture system in *vitro* were determined by SABC as described previously [9]. Cells plated on slides were untreated (control group, equal RPMI-1640 or ECGM solution) or treated with an 1/3 IC_50_ dose of NCTD (NCTD group), and primary antibody of Bcl-2 (rabbit polyclonal antibody, 1:50, Santa Cruz, USA), biotinylated secondary antibody (goat anti-rabbit IgG, 1:100), SABC reagents and DAB solution (all from Boster Co., China) were in turn added. Then, slides were rinsed, dehydrated, mounted and observed under a microspectrophotometer (Leitz Dmrbe). For negative control, the slides were treated with PBS in place of primary antibody. Ten sample slides in each group were chosen for analysis. The positive index of Bcl-2 represented expression of Bcl-2 protein.

### Migration assay *in vitro*

Transwell migration chambers i.e., 24-well plates by Transwell chambers with polycarbonate filters (pore size 8 μm, diameter 6.5 mm) were used in this assay. HT-29 cells (1 × 10^5^ cells/ml) or HDLECs (5 × 10^4^ cells/ml) were inoculated in the upper compartment of the chamber (200 μl/chamber), 0.8 ml RPMI-1640 medium with 10 % FBS or ECGM medium was added to the lower compartment of the chamber (200 μl/chamber). For the co-culture system, HDLECs were added to the upper compartment of the chamber (200 μl/per chamber), HT-29 cells to the lower compartment of the chamber (200 μl/per chamber) in 0.8 ml of RPMI-1640 medium with 10 % FBS. Cells were untreated (control group, equal ECGM solution) or treated with above 1/3 IC_50_ NCTD (NCTD group; 18.7 μg/ml for HT-29 cells, 2.5 μg/ml for HDLECs, 5.3 μg/ml for co-culture), 100 μl mF4-31C1 (mF4-31C1 group) and NCTD+ mF4-31C1 (NCTD + mF4-31C1 group) (6 chambers/per group), respectively, in fresh culture medium (chambers/per group) at 37 °C in 5 % CO_2_ for 24 h. Total number of migrating cells were measured and counted in five independent microscopic visual fields (×100) under an inverted microscope (Nikon TS100, Japan), and expressed as mean number per one field. Each experiment was performed thrice.

### Invasion assay *in vitro*

Matrigel invasion chamber i.e. 24-well plates by Transwell chambers with polycarbonate filters (pore size 8 μm, diameter 6.5 mm) coated on the upper side with Matrigel (Becton Dickinson were used in this assay. HT-29 cell (1 × 10^5^ cells/ml) or HDLEC (5 × 10^4^ cells/ml) suspensions were transferred to the upper compartment of the chamber (200 μl/every chamber), while 0.8 ml RPMI-1640 medium with 10 % FBS or ECGM medium was added to the lower compartment of the chamber. For the co-culture system, HDLECs were added to the upper compartment of the chamber, HT-29 cells to the lower compartment of the chamber (200 μl/every chamber) in 0.8 ml of RPMI-1640 medium with 10 % FBS. Cell experiment was performed as above migration assay. The number of invading cells through the filter was counted after H&E staining and plotted as the mean number of invading cells per optic field in three independent experiments.

### Determination of VEGF-A, VEGF-C, VEGF-D, VEGFR-2, VEGFR-3 *in vitro* and *in vivo*

The expression of VEGF-A, VEGF-C, VEGF-D, VEGFR-2 and VEGFR-3 at protein and mRNA levels from the 3-D culture of HDLECs or the co-culture system *in vitro*, and the *in-situ* xenografts *in vivo* were determined by S-P staining, western blotting and fluorescent quantitative RT-PCR as described previously [23, 47].

For S-P staining, slides were treated according to the kit brochure (Jinmei Biotechnology Co., Ltd., Shanghai), added in order with primary antibody [rabbit anti-human monoclonal antibody VEGF-A (Santa Gruz), VEGF-C (Invitrogen), VEGF-D (Abcam, USA), VEGFR-2 (Cell Signaling, USA), VEGFR-3 (Cell Signaling), biotinylated anti-rabbit secondary, HRP logo Streptavidin and DAB solution, respectively. Then, slides were rinsed, dehydrated, mounted and observed under an optic microscope (Olympus, Japan). For negative control, the slides were treated with PBS in place of primary antibody. Six sample slides in each group were chosen by analysis. Visual fields (>10) were observed or >500 cells were counted per slide.

Lowry method protein kit (Puli Lai Co., Shanghai) were used for western blotting according to the kit brochure. An aliquot of 20 mg of proteins was subjected to sodium dodecyl sulfate-polyacrylamide gel electrophoresis (SDS-PAGE), and transferred to a PVDF membrane. One hour after being blocked with PBS containing 5 % non-fat milk, the membrane was incubated overnight, was then added in order with each primary antibody [anti-VEGF-A, anti-VEGF-C, anti-VEGF-D (Abcam), and anti-VEGFR-2, anti-VEGFR-3, anti-β-actin (Cell Signaling)], HRP-labeled secondary antibody (Abcam) (all 1:1000)], HistoFine (Dako, Glostrup, Denmark) for 2 h. The target proteins were visualized by an enhanced chemiluminescent reagent (GE Healthcare, USA), imaged on the Bio-Rad chemiluminescence imager. The gray value and gray coefficient ratio of each protein was analyzed and calculated.

Fluorescent quantitative RT-PCR was performed as described by the manufacturer. Total RNA was extracted using the TRIzol reagent (Invitrogen). The primers for amplification were designed and synthesized by Sangon Co., Shanghai. The primers for VEGF-A, VEGF-C, VEGF-D, VEGFR-2, VEGFR-3 and GAPDH *in vitro* and *in vivo* were as shown in Table 1. RT-PCR reaction conditions and the amplifying conditions *in vitro* were as described previously [23]. GAPDH was used as an internal control, with the annealing temperature of 56 °C for 40 cycles (94 °C for 5 min, 94 °C for 30 s, 55 °C for 30 s, 72 °C for 30 s, and 72 °C for 10 min). PCR products (10 μl) were placed onto 15 g/L agarose gel and observed by ethidium bromide staining using the ABI PRISM 7300 SDS software. The relative mRNA expression levels was calculated by the formula (relative mRNA expression = 2^-**△△**Ct^).

### Statistical analysis

Statistical analyses were performed using SPSS 13.0 and Microsoft Excel Office 2007 for Windows. All data were presented as mean ± SD. Statistical differences were evaluated using Student’s *t* test or the Chi-square test. *P* < 0.05 was considered statistically significant. Survival curves were calculated with the Kaplan-Meier method and were compared using the log-rank test.

## Results

### NCTD inhibits growth of the in-situ colonic xenografts *in vivo*

**Fig. 1:**
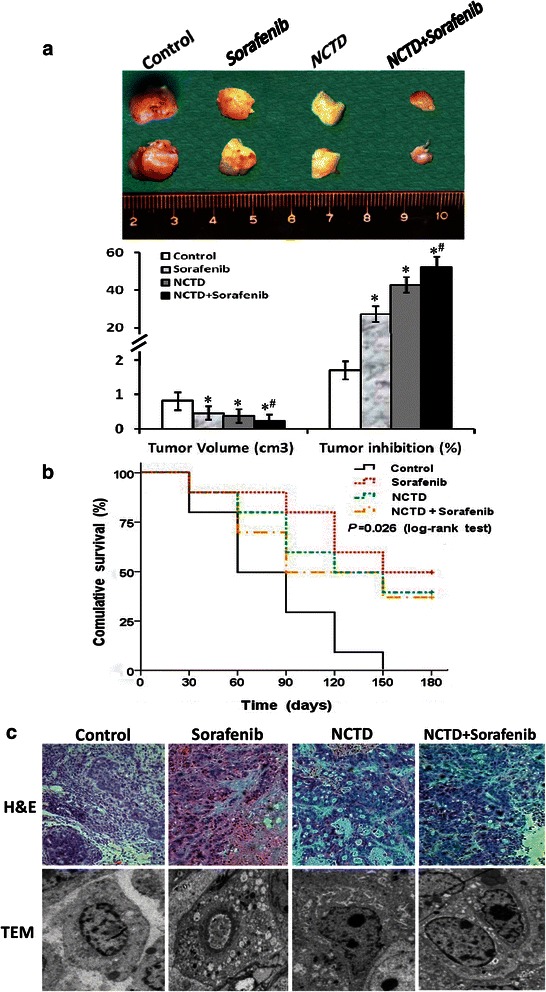
NCTD inhibits growth of the *in-situ* colonic xenografts and prolongs survival time of the xenograft mice *in vivo*. **a** Tumor growth of the *in-situ* colonic xenografts of each group. A pink, pale, fish-like, round or oval *in-situ* xenograft was found at intestinal wall at the 6th week end, with average tumor volume of 818.45 ± 53.16 mm^3^ in control group; but the size and volume of the xenograft in Sorafenib, NCTD or NCTD + Sorafenib group were decreased significantly (*P < 0.001), with increased tumor inhibition rate (^#^*P* < 0.0001) as compared to control group, and a significant tumor inhibition in NCTD + Sorafenib group in comparison with Sorafenib or NCTD group (^**§**^*P* < 0.01). **b** Kaplan-Meier survival curves for the xenograft mice of each group. A prolonged survival time was observed in Sorafenib, NCTD or NCTD + Sorafenib group as compared to control group (log-rank test, *P* = 0.026). **c** The histomorphologic structure of the *in-situ* colonic xenografts of each group (H&E, magnification × 200; TEM, magnification × 6000). In control group, colonic wall structure was destroyed, tumor cells showed infiltrative growth or arranged in clusters funicular i.e. cancer nests, with abundant cytoplasm, deep dyeing nucleus, increased mitotic phase and connective tissue among tumor cells under an optic microscope (C_H__&__E_); irregular tumor cells with abundant microvilli, clear organelles and chromatin enrichment under a TEM (C_TEM_). But in Sorafenib, NCTD or NCTD + Sorafenib group, tumor cells, cancer nests, different-sized glands and part of blood vessels tissues were destroyed; many destroyed, even apoptotic tumor cells, part of vacuolar degeneration were observed (C_H__&__E_); also, disappearing microvilli, mitochondrial swelling, golgiosome atrophy, vacuolar degeneration, nuclear shrinkage, chromatin aggregation, chromosome condensation, and typical apoptotic bodies were found (C_TEM_)

We previously reported that NCTD has multiple antitumor activities against different tumor cells [9, 12, 51, 61, 63], whereas Sorafenib is an oral multi-kinase inhibitor that blocks proliferation and carcinogenesis of different tumor cells including colonic adenocarcinoma cells by a dual mechanism including targeting several receptor tyrosine kinases such as VEGFR-2 and VEGFR-3 [37, 38]. Here, we investigated the antitumor activity of NCTD for HCACs *via* tumor assays of the *in-situ* colonic xenografts and a survival analysis of xenograft mice *in vivo*. In control group, pink or pale, round or oval *in-situ* xenografts appeared gradually at colonic wall of nude mice about 6 weeks after subcutaneous xenograft of HT-29 cells was inserted into the concave niche of the cecum, with average tumor volume 818.45 ± 53.16 mm^3^ (Fig. 1a). And, it was observed in the *in-situ* xenografts with H&E staining under an optic microscope that colonic wall structure was destroyed, tumor cells showed infiltrative growth or arranged in clusters funicular i.e. cancer nests, with abundant cytoplasm, deep dyeing nucleus, increased mitotic phase, and connective tissue among tumor cells (Fig. 1 c_H__&__E_); irregular tumor cells with abundant microvilli, clear organelles and chromatin enrichment under a TEM (Fig. 1 c_TEM_). But in NCTD, Sorafenib or NCTD + Sorafenib group, the *in-situ* xenograft volume was markedly decreased, with an increased tumor inhibitory rate (Fig. 1a; *P* < 0.001, or *P* < 0.0001) as compared to control group, and more obvious tumor inhibition in NCTD + Sorafenib group in comparison with Sorafenib or NCTD group (Fig. 1a, *P* < 0.01); it was also found that tumor cells, different-sized glands and part of blood vessels were destroyed, many destroyed, even apoptotic tumor cells and part of vacuolar degeneration were observed (Fig. 1 c_H__&__E_); disappearing microvilli, mitochondrial swelling, golgiosome atrophy, organelle vacuoles, nuclear shrinkage, chromatin aggregation, chromosome condensation and typical apoptotic bodies were seen (Fig. 1 c_TEM_). And, it is comforting that xenograft mice of each group were all alive at the end of the experiments, and that survival time in Sorafenib, NCTD or NCTD + Sorafenib group was significantly prolonged as compared to control group (log-rank test, *P* = 0.026; Fig. 1b). Thus, we believed that NCTD or in combination with Sorafenib inhibits growth of the *in-situ* colonic xenografts effectively and safely *in vivo*.

### NCTD inhibits tumor lymphangiogenesis and lymphatic micrometastasis of the in-situ colonic xenografts *in vivo*

**Fig. 2:**
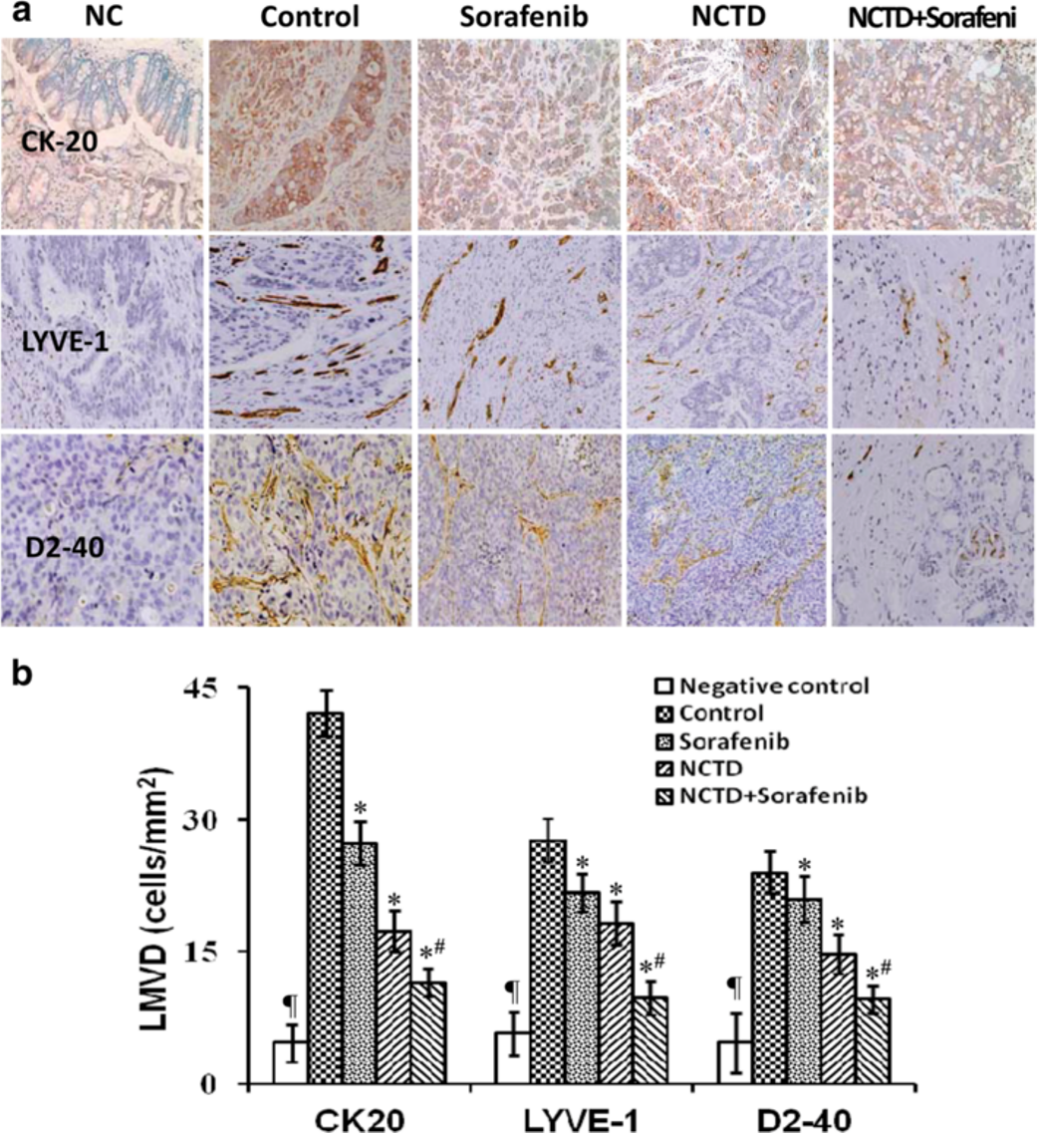
NCTD inhibits tumor lymphangiogenesis and lymphatic micrometastasis of the *in-situ* colonic xenografts by immunohistochemistry *in vivo*. **a** The expression of CK-20, LYVE-1 and D2-40 protein products of the *in-situ* colonic xenografts of each group (SABC, magnification × 200); NC, negative control, with only IgG to rule out the non-specific HRP-activated signal. **b** The LMVD of the *in-situ* colonic xenografts of each group. The lowest LMVD, with no or weaken expression of CK20, LYVE-1 or D2-40s in NC group (*P* < 0.001, *vs*. control, Sorafenib, NCTD or NCTD + Sorafenib group); the lower LMVD, with weaken expression of CK20, LYVE-1 or D2-40 and few, thin and destroyed microvessels in Sorafenib, NCTD or NCTD + Sorafenib group as compared with control group (all *P < 0.05). Of them, the LMVD of NCTD + Sorafenib group was lowest (^*#*^*P* < 0.001, *vs*. Sorafenib or NCTD group)

**Fig. 3:**
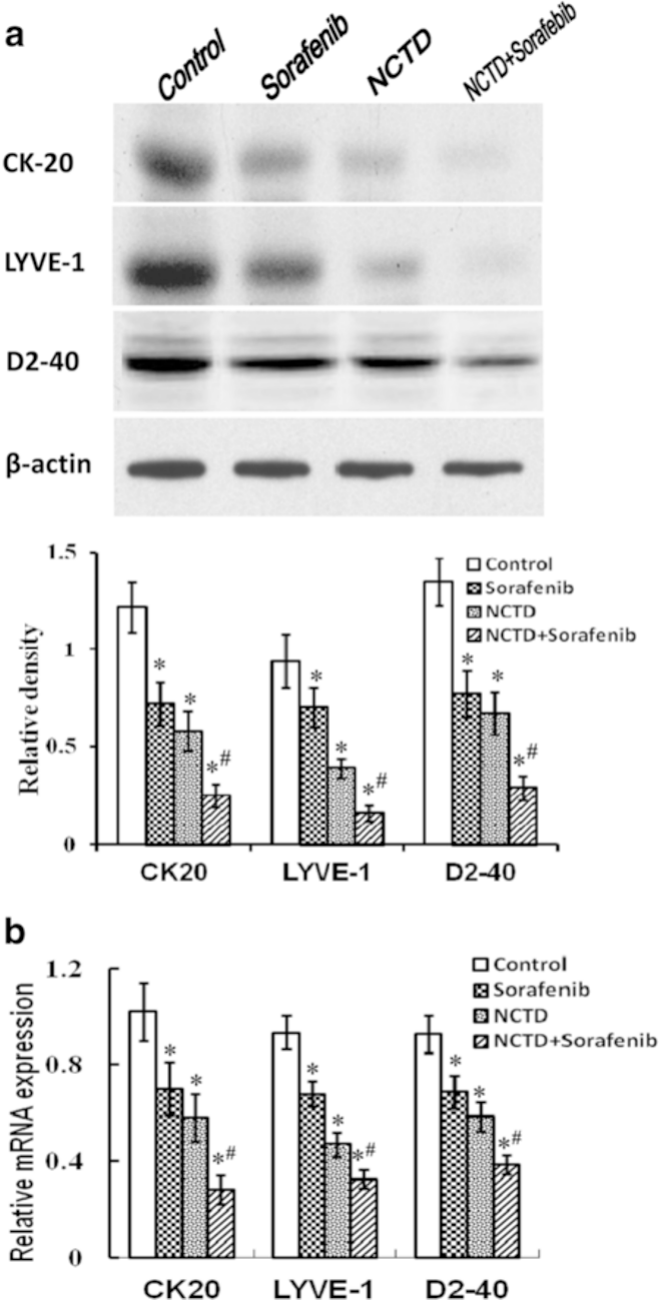
NCTD inhibits tumor lymphangiogenesis and lymphatic micrometastasis of the *in-situ* colonic xenografts by western blotting and RT-PCR *in vivo*. **a** The expression of CK-20, LYVE-1 and D2-40 proteins in the *in-situ* colonic xenografts of each group (western blotting): expression of CK-20, LYVE-1 and D2-40 proteins in NCTD, Sorafenib or NCTD + Sorafenib group was decreased significantly as compared to control group (*P < 0.05), with the lowest expression of these proteins in NCTD + Sorafenib group (^**#**^*P* < 0.001). **b** Fluorescent quantitative RT-PCR: the expression of CK-20, LYVE-1 and D2-40 mRNAs was also decreased significantly in all experimental groups as compared to control group (*P < 0.05); and the expression of CK-20 or LYVE-1 mRNAs in NCTD + Sorafenib group was significantly lower than those of NCTD or Sorafenib group (^**#**^*P* < 0.001)

Tumor lymphangiogennesis plays an important role in promoting tumor growth and metastasis *via* the lymphatic [25, 31, 43]. To verify the antitumor lymphangiogenic activity of NCTD, in the experiment, we determined lymphatic specific marker - LYVE-1, D2-40 and lymphatic micrometastic marker - CK20, and their LMVD of the *in-situ* colonic xenografts. In control group, some dense, thin wall, large lumen, tubular or irregular microvessels with strong brown positive staining in cytoplasm or cytomembrane, in line with the morphological features of lymphatic capillaries, were visualized. While weaken expression of CK20, LYVE-1 and D2-40 protein products, with little brown tan vessels with rebirth tumor cells, invaded and destroyed microvessel profile among apoptotic tumor cells (Fig. 2a). And lower LMVD were observed in NCTD, Sorafenib or NCTD + Sorafenib group as compared to control group (all *P* < 0.05), with the lowest LMVD in NCTD + Sorafenib group (*P* < 0.001) (Fig. 2b). Furthermore, the expression of CK20, LYVE-1 and D2-40 at protein and mRNA levels of the *in-situ* colonic xenografts in NCTD, Sorafenib, or NCTD + Sorafenib group were significantly decreased when compared with control group (all *P* < 0.05), with the lowest CK20, LYVE-1 or D2-40 expression in NCTD + Sorafenib group (*P* < 0.001) (Fig. 3), which was in line with above immunohistochemical detection. It was showed that NCTD or Sorafenib inhibited the expression of CK20, LYVE-1 and D2-40 proteins/mRNAs, decreased the LMVD of the *in-situ* colonic xenografts *in vivo*. So, we believed that NCTD or in combination with Sorafenib inhibits tumor lymphangiogenesis and lymphatic micrometastasis of the *in-situ* colonic xenografts *in vivo*, thus verified the antitumor lymphangiogenic activity of NCTD.

### NCTD inhibits lymphatic tube formation of HDLECs and co-culture *in vitro*

**Fig. 4:**
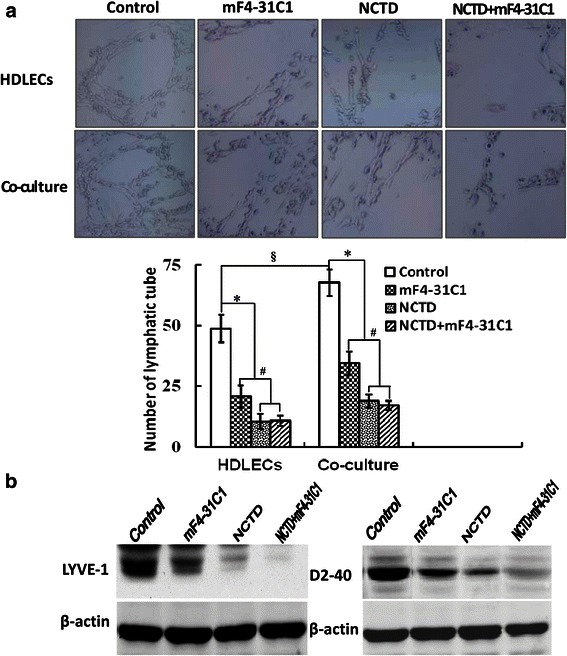
NCTD inhibit lymphatic tube formation of the 3-D culture of HDLECs or the 3-D co-culture system *in vitro*. **a** Capillary-tube formation and capillary-tube number of each group under an inverted light microscope (magnification × 200). When seeded on the lower compartment of the chamber coated with Matrigel matrix for one week, HDLECs formed typical capillary-like tubes with pipe wall, the lumen and progressive branches, while the capillary tube formation was more obvious in the upper compartment of the chamber with HT-29 cells than without (^**§**^*P* < 0.01). After treatment with NCTD, mF4-31C1 or NCTD+ mF4-31C1, HDLECs didn’t form above capillary-like tube, with visible cell aggregation, float, nuclear fragmentation, apoptosis; and the capillary-tube number in these groups was markedly decreased as compared to control group (*P < 0.000), while this number in NCTD or NCTD+ mF4-31C1 group was less than that of mF4-31C1 group (all ^#^*P* < 0.01). **b** The expression of LYVE-1 and D2-40 from the 3-D co-culture system *in vitro* using western blotting. The positive expression of LYVE-1 and D2-40 proteins was observed in control group; but LYVE-1, D2-40 protein expression was significantly downregulated in mF4-31C1, NCTD or NCTD + mF4-31C1 group (^#^*P* < 0.01)

Lymphatic tube formation is referred to as a critical step for lymphangiogenesis and tumor lymphangiogenesis [23, 25, 31, 43]. To further verify the anti-lymphangiogenic activity of NCTD, we observed the lymphatic capillary-like structures (i.e., lymphangiogenesis) formed from the 3-D culture of HDLECs and the co-culture system consisting of HT-29 cells and HDLECs *in vitro* and their LYVE-1, D2-40 expression, by using a soluble VEGFR-3 antibody with antilymphangiogenesis activity mF4-31C1 as experiment control. As shown in Fig. 4a, when seeded on the lower compartment of the chamber coated with Matrigel matrix for 24 h, HDLECs started to paste the well wall, grew, spread out, formed the cell groups composed of multangular or pseudopod cells; formed typical capillary-like tubes with pipe wall, the lumen and progressive branches after 1 week, while the capillary tube formation was more obvious in the co-culture system than alone HDLEC culture, showing that HT-29 cells promoted capillary tube formation of HDLECs in the co-culture system. After treatment with NCTD, mF4-31C1 or NCTD + mF4-31C1, HDLECs didn’t form above capillary-like tube structures, with visible cell aggregation, float, nuclear fragmentation and apoptosis. Moreover, the number of the capillary-like tubes in NCTD, mF4-31C1 or NCTD + mF4-31C1 group was markedly decreased as compared to control group (*P* < 0.000), while the capillary tube number in NCTD or NCTD + mF4-31C1 group was less than that of mF4-31C1 group (*P* < 0.01). In order to identify if these capillary-like tubes are lymphatic capillary tubes, LYVE-1 and D2-40 in HDLECs and the co-culture system were determined using western blotting. As shown in Fig. 4b, the positive expression of LYVE-1 and D2-40 proteins was observed in the capillary-like tubes formed from the 3-D culture of HDLECs or the co-culture system in control group, and expression of LYVE-1 and D2-40 in the co-culture system was markedly up-regulated than alone HDLEC culture, identifying that HT-29 cells promoted lymphatic tube formation of HDLECs in the co-culture system; but LYVE-1, D2-40 expression was significantly downregulated in NCTD, mF4-31C1 or NCTD + mF4-31C1 group as compared to control group (*P* < 0.01). The results implicated that NCTD, the same as mF4-31C1, inhibited the lymphatic tube formation from the 3-D culture of HDLECs and the co-culture system *in vitro*, while this effect of NCTD or NCTD + mF4-31C1 was stronger. Collectively, NCTD inhibits the lymphatic tube formation of HDLECs and the tumor lymphangiogenesis of HCACs *in vitro*, thus further verify the anti-lymphangiogenic activity of NCTD.

### NCTD affects malignant phenotypes of HDLECs and co-culture *in vitro*

**Fig. 5:**
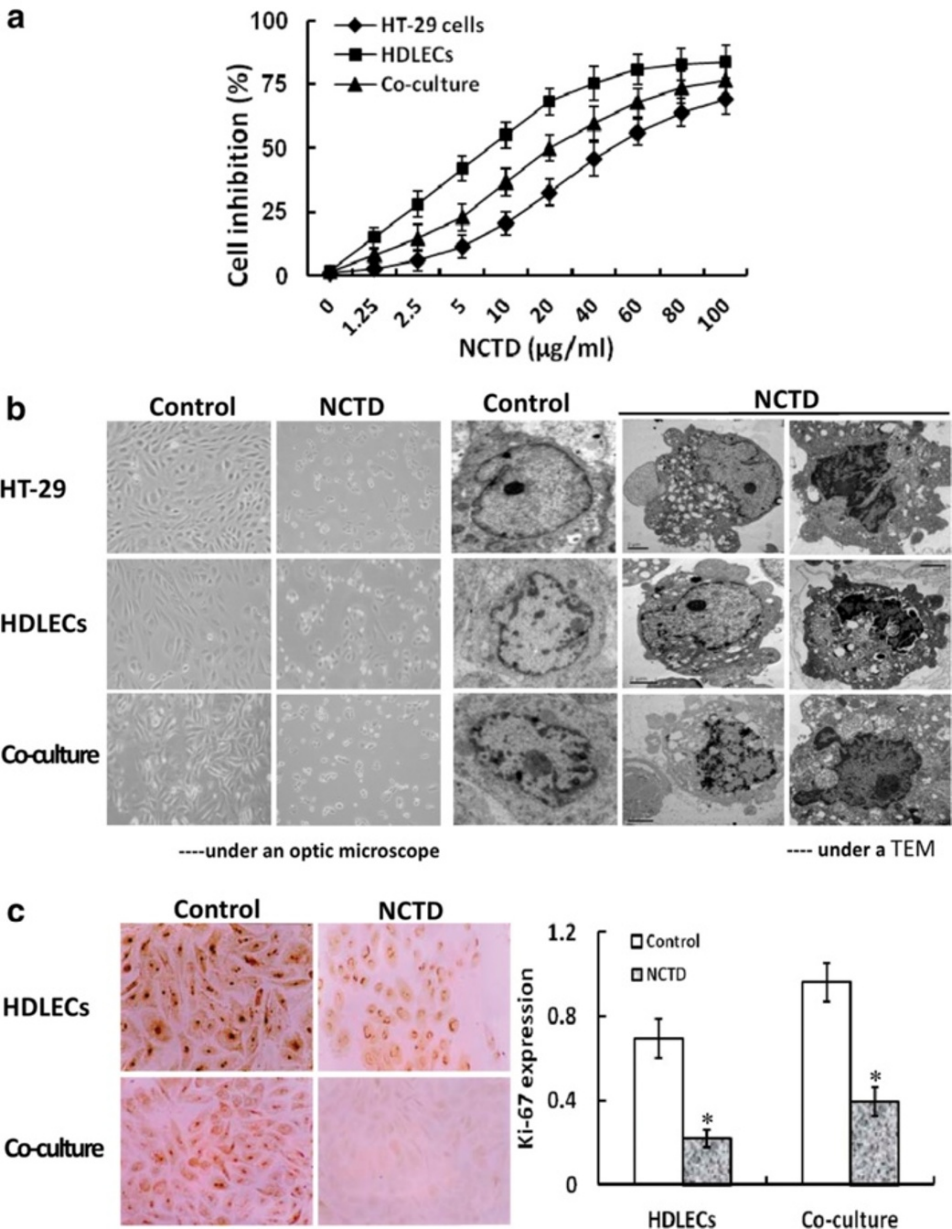
NCTD inhibits proliferation of HT-29 cells, HDLECs and the co-culture system *in vitro*. **a** The dose–response curves of NCTD effect on HT-29 cells, HDLECs and the co-culture system with IC_50_ value 56.8 μg/ml for HT-29 cells, 6.8 μg/ml for HDLECs and 15.8 μg/ml for the co-culture system. Cell number was counted by the MTT method. **b** Histomorphologic of HT-29 cells, HDLECs and the co-culture system under an inverted optic microscope (magnification × 200) and a TEM (magnification × 8000): predominantly shuttle-shape cells, with abundant cytoplasm, clear nuclei, and abundant microvillus, clear organelles, larger nucleus cytoplast ratio, irregular nuclei and chromatin enrichment in control group; after treatment with 1/3 IC_50_ NCTD for 24 h, visible cell aggregation, float, nuclear shrinkage, chromosome condensation, microvillus decreasing, golgiosome atrophy, mitochondria swell, cytoplast vacuole, nuclear fragmentation, chromatin aggregation and typical apoptotic bodies, or even death. **c** The inhibitory effect of NCTD on expression of proliferating marker Ki-67 in HDLECs and the co-culture system *in vitro*. The positive expression, with brown-yellow dye, of Ki-67 protein product occurred in cell nucleoli. After treatment with 1/3 IC_50_ NCTD for 48 h, the positive index of Ki-67 expression in HDLECs (0.696 ± 0.0611 *vs*. 0.221 ± 0.042) or the co-culture system (0.964 ± 0.098 *vs*. 0.397 ± 0.068) was respectively decreased significantly as compared to control group (all *P* < 0.001), and the dye of cell nucleoli became light and shallow

**Fig. 6:**
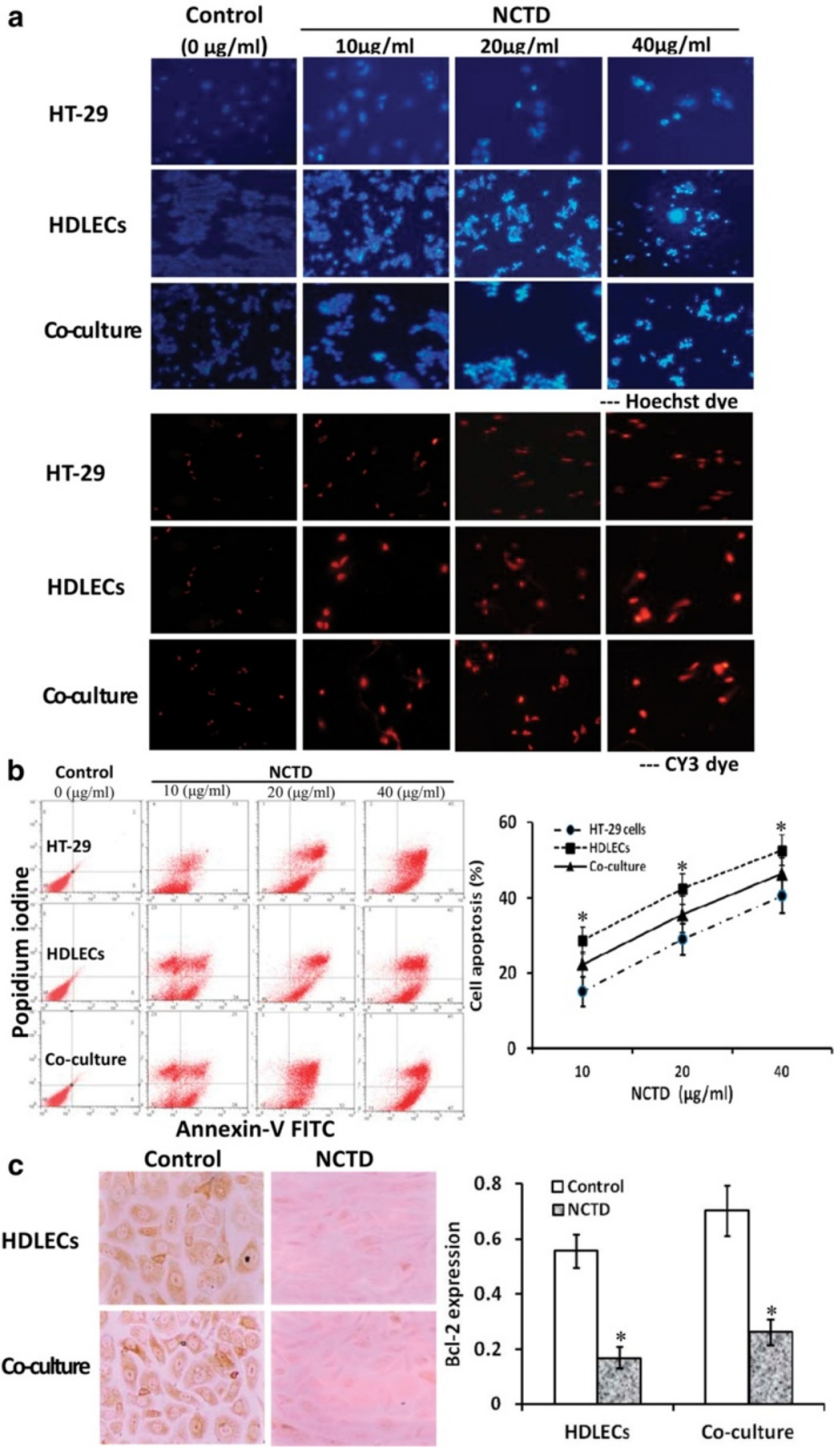
NCTD induces apoptosis of HT-29 cells, HDLECs and the co-culture system *in vitro*. **a** Hoechst and CY3 immunofluorescent dyes (fluorescent microscope, magnification × 200). No apoptotic cells were observed in control group (0 μg/ml of NCTD). After treatment with NCTD for 24 h and with concentration increasing, apoptosis which presented bright blue/brown dyes even with fragment nuclei of HT-29 cells, HDLECs or the co-culture system was increased significantly as compared to control group; of them, fewer apoptosis which showed more shallow blue or brown dye in HT-29 cells or the co-culture system as compared to HDLECs. **b** Cell apoptosis induced by different concentration of NCTD and time-response curves *via* FCM. In FCM scatter diagram, the left lower quadrant shows live cells (FITC-/PI-), the right upper quadrant for necrotic or death cells (FITC+/PI+), and the right lower quadrant for apoptotic cells (FITC+/PI-)]. No apoptotic cells were observed in control group (0 μg/ml of NCTD). After treatment with NCTD for 24 h and with concentration increasing, apoptotic percent of HT-29 cells, HDLECs or the co-culture system was increased significantly in a time-dependent manner, and with decreased living HT-29 cells, HDLECs and co-culture cells, increased apoptotic cells under the right lower quadrant (FITC+/PI-), more necrotic or death cells under the right upper quadrant (FITC+/PI+) as compared to control group (*P <* 0.001); moreover, apoptotic percent of HT-29 cells or the co-culture system was less than that of HDLECs (*P < 0.05). **c** The inhibitory effect of NCTD on expression of anti-apoptotic gene Bcl-2 in HDLECs and the co-culture system *in vitro*. The positive expression, with brown-yellow dye, of Bcl-2 protein product occurred in cytoplasm. After treatment with 1/3 IC_50_ NCTD for 48 h, the positive index of Bcl-2 protein expression in HDLECs (0.556 ± 0.066 *vs*. 0.108 ± 0.039) or the co-culture system (0.702 ± 0.098 *vs*. 0.221 ± 0.048) was respectively decreased significantly as compared to control group (all *P* < 0.001), and the dye of cell nucleoli became light and shallow

**Fig. 7:**
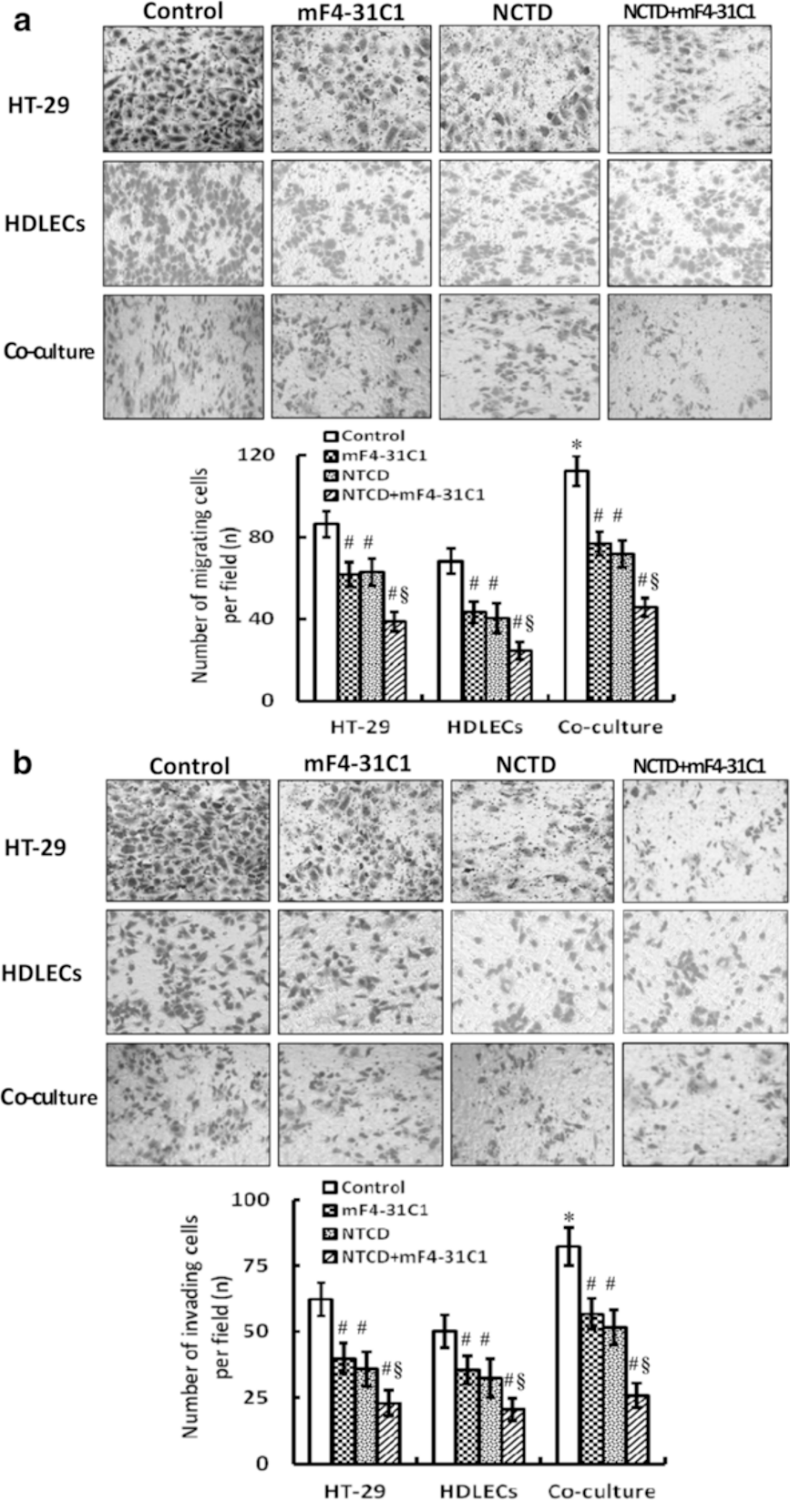
NCTD inhibits migration and invasion of HT-29 cells, HDLECs and the co-culture system *in vitro* (i**nverted** optic microscope, magnification × 200). **a** Migration assay by Transwell migration chambers: total number of migrating cells of the co-culture system was more than that of HT-29 cells or HDLECs (all *P < 0.01) in control group. After treatment with NCTD, mF4-31C1 or NCTD+ mF4-31C1, total number of migrating cells in these groups was decreased significantly as compared to control group (all ^**#**^*P* = 0.000); of them, the number of migrating cells in NCTD+ mF4-31C1group was the least (^**§**^*P* = 0.01). **b** Invasion assay by Matrigel invasion experiment: total number of invading cells through the filter coated Matrigel of the co-culture system was also more than that of HT-29 cells or HDLECs (all*P < 0.01) in control group; the number of invading cells in NCTD, mF4-31C1 or NCTD + mF4-31C1 group was decreased significantly as compared to control group (all ^**#**^*P* = 0.000), with the least invading cells in NCTD + mF4-31C1group (^**§**^*P* = 0.01)

Proliferation, apoptosis, migration and invasion of the cells are referred to as critical early steps for lymphangiogenesis [23, 25, 31, 43]. To confirm anti-lymphangiogenic activity of NCTD, we further observed the effects of NCTD on malignant phenotypes i.e. proliferation, apoptosis, migration and invasion of HT-29 cells, HDLECs and the co-culture system. As shown in Fig. 5a and b, the cultured HT-29 cells and HDLECs began to growth at 8th hour, maturated at one day, being predominantly of shuttle-shape, or accumulation, with abundant cytoplasm, clear nuclei; of them, cell proliferation and growth of the co-culture system was more active than those of alone HDLEC culture; after NCTD treatment, a significant inhibition of proliferation of HT-29 cells, HDLECs and the co-culture system as compared to control group was showed in a dose-dependent manner with the NCTD IC_50_ value 56.18 μg/ml for HT-29 cells, 6.8 μg/ml for HDLECs and 15.8 μg/ml for the co-culture system; and the morphology of HT-29 cells, HDLECs and the co-culture system showed visible cell aggregation, float, nuclear condensation or fragmentation, cataclysm. In addition, proliferating marker Ki-67 of LYVE-1 or D2-40-positive HDLECs and co-culture system in *vitro* were determined by SABC method in order to further observe the inhibitory effect of NCTD on proliferation of HDLECs and the co-culture system. As shown in Fig. 5c, after treatment with 1/3 IC_50_NCTD for 48 h, the positive index of Ki-67 expression in HDLECs or the co-culture system was decreased significantly as compared to control group (HDLECs: 0.69 ± 0.0611 *vs*. 0.221 ± 0.042, co-culture: 0.964 ± 0.098 *vs*. 0.397 ± 0.068; all *P* < 0.001), and the dye of cell nucleoli became light and shallow. Moreover, these observations were confirmed by some apoptotic assays *via* immunofluorescent dyes (Fig. 6a), FCM (Fig. 6b, Table 2), apoptotic-related gene *via* immunohistochemistry (Fig. 6c), and microstructure observation under TEM (Fig. 5b), which revealed that apoptotic percent of HT-29 cells or the co-culture system was less than that of HDLECs (*P <* 0.05), anti-apoptotic gene Bcl-2 expression of the co-culture system was higher than that of HDLECs (*P <* 0.05) in control group; NCTD induced S-phase cell cycle arrest (*P* < 0.001) and cell apoptosis in a dose-, time-dependent manner, and inhibited apoptotic-related gene expression (*P* < 0.001), i.e., apoptotic cells [bright blue/brown dye cells by immunofluorescent dyes; total cells under right lower/upper quadrant of cells (FITC+/PI-, FITC+/PI+) by FCM] were increased, the positive index of anti-apoptotic gene Bcl-2 expression was decreased significantly, with lighter and more shallow dye of cell nucleoli, as compared to control group (*P* < 0.001) (Fig. 6, Table 2); and, microvillus decreasing, cytoplast vacuole, nuclear shrinkage, chromatin aggregation or condensation, and typical apoptotic bodies in NCTD group were observed under a TEM (Fig. 5b). Furthermore, as shown in Fig. 7, total number of migrating cells and total number of invading cells through the filter coated Matrigel of HT-29 cells or the co-culture system were more than those of alone HDLECs (all *P < 0.01) in control group; after treatment, total number of migrating or invading cells in NCTD, mF4-31C1 or NCTD+ mF4-31C1 group was decreased as compared to control group (all ^**#**^*P* = 0.000); of them, the number of migrating or invading cells in NCTD + mF4-31C1group was the least (^**§**^*P* = 0.01). It is shown that HT-29 cells may promote proliferation, migration and invasion of HDLECs in the co-culture system and decrease cell apoptosis *in vitro*; while NCTD inhibited proliferation, invasion and migration of not only HT-29 cells but also HDLECs and the co-culture system, and induced apoptosis of these cells *in vitro* in a dose or time dependent manner; and NCTD in combination with mF4-31C1 had stronger antitumor effect. Taken together, these *in vitro* results indicated that NCTD alone or in combination with mF4-31C1 inhibited the lymphatic tube formation of HDLECs and the tumor lymphangiogenesis of HCACs by affecting these malignant phenotypes, inhibiting the expression of proliferating marker Ki-67 and anti-apoptotic gene Bcl-2 and induced S-phase cell cycle arrest, thus confirmed the anti-lymphangiogenic activity of NCTD.

**Table 2:** Effect of NCTD on cell cycle distribution of HT-29, HDLECs and Co-culture system (mean ± SD, %)

Groups	HT-29		HDLECs		Co-culture	
	G_2_/M	S	G_2_/M	S	G_2_/M	S
Control	25.5 ± 3.3	21.7 ± 3.1	16.5 ± 2.3	16.7 ± 2.3	21.5 ± 3.3	18.9 ± 3.0
NCTD (IC_50_)	28.7 ± 4.8	9.3 ± 1.8*	15.7 ± 2.8*	6.3 ± 1.2*	22.7 ± 2.8*	7.8 ± 1.6*

*P < 0.001, vs. control group

### NCTD downregulates expression of VEGF-A, VEGF-C, VEGF-D, VEGFR-2 and VEGFR-3 *in vitro* and *in vivo*

**Fig. 8:**
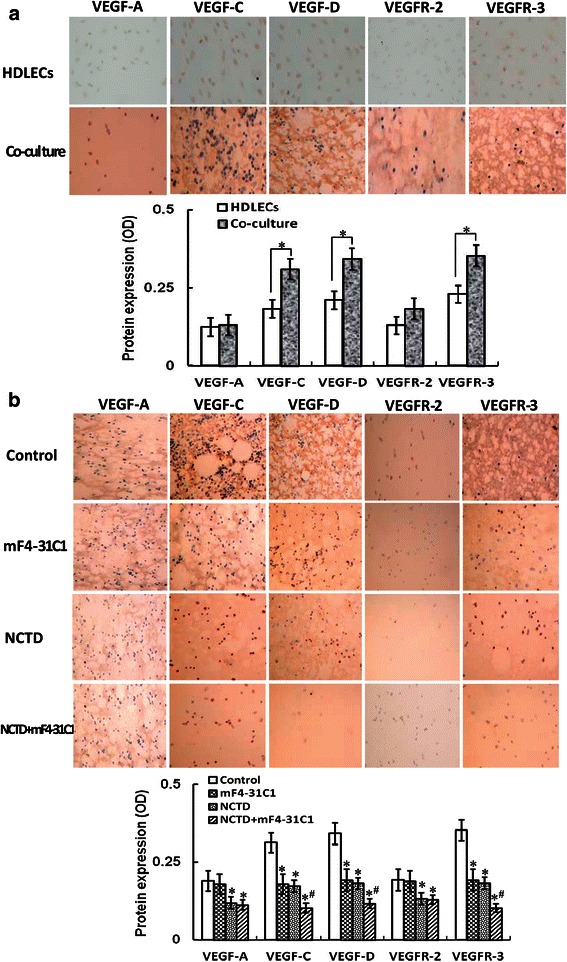
The expression of VEGF-A, VEGF-C, VEGF-D, VEGFR-2 and VEGFR-3 protein products of HCACCs and the co-culture system of each group and the effect of NCTD on expression of these protein products *in vitro* (S-P staining, magnification × 200). **a** The expression of VEGF-A, VEGF-C, VEGF-D, VEGFR-2 and VEGFR-3 protein products of HCACCs and the co-culture system of each group. The expression of VEGF-C, VEGF-D and VEGFR-3 protein products (brown staining in cytoplasm) of the co-culture system was higher than those of alone HCACC culture (*P = 0.001); but there is no difference on the expression of VEGF-A and VEGFR-2 protein products between alone HCACC culture and the co-culture system. **b** The inhibitory effect of NCTD on expression of these protein products of the co-culture system. The expression of VEGF-C, VEGF-D and VEGFR-3 protein products in NCTD, mF4-31C1 or NCTD + mF4-31C1 group was downregulated significantly as compared to control group (*P < 0.01), and the expression of these proteins in NCTD + mF4-31C1 group was lower than that of NCTD or mF4-31C1 group (^**#**^*P* < 0.05); whereas the expression of VEGF-A and VEGFR-2 protein products in NCTD or NCTD + mF4-31C1 group was downregulated significantly as compared to control or mF4-31C1 group (*P < 0.01), but no difference on VEGF-A and VEGFR-2 expression between control group and mF4-31C1 group

**Fig. 9:**
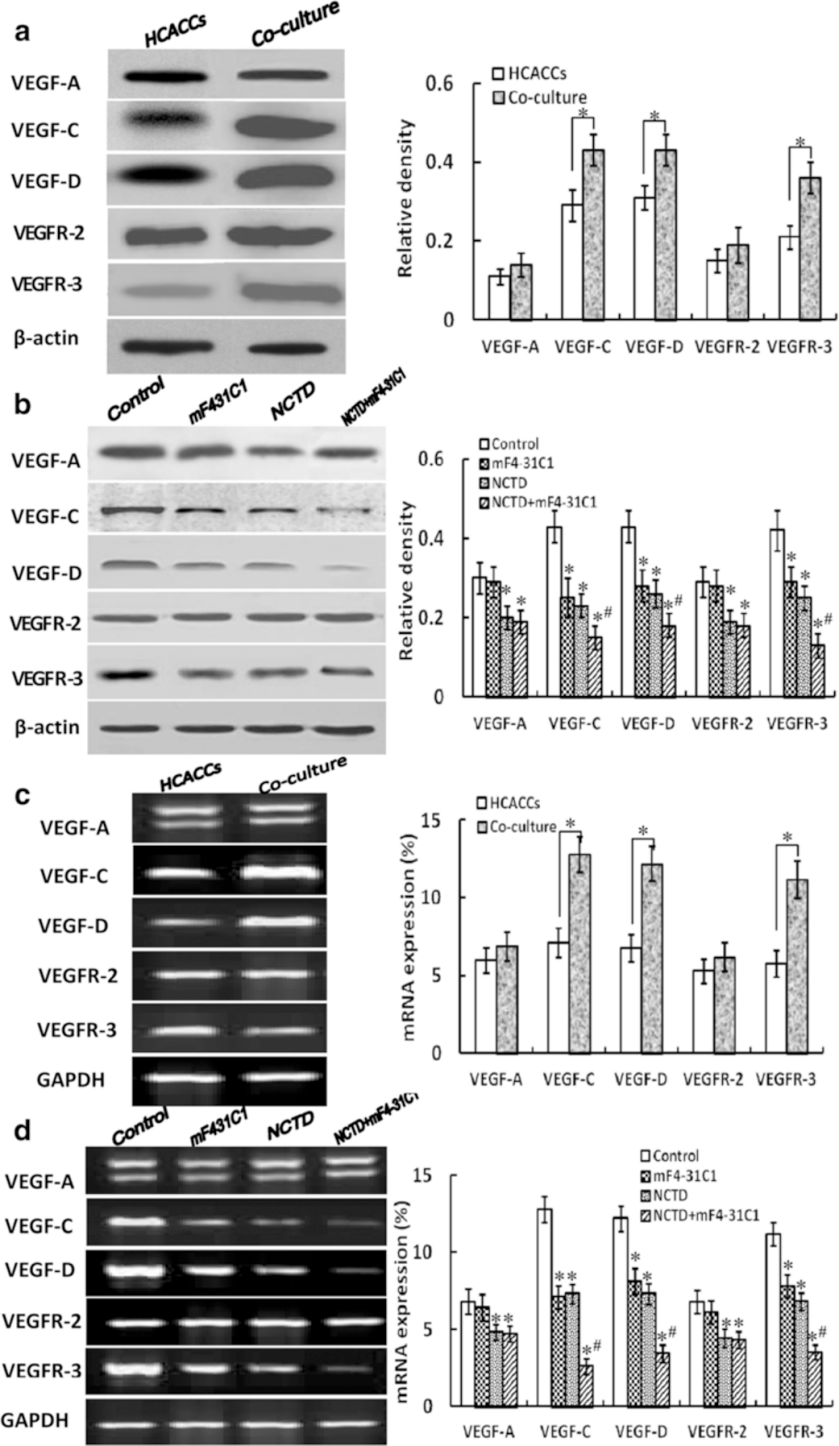
The expression of VEGF-A, VEGF-C, VEGF-D, VEGFR-2 and VEGFR-3 proteins/mRNAs of HCACCs and the co-culture system and the effect of NCTD on expression of these proteins/ mRNAs by western blotting (**a** and **b**) and fluorescent quantitative RT-PCR (**c** and **d**) *in vitro*. **a** and **b** Protein expression by western blotting: the expression of VEGF-C, VEGF-D and VEGFR-3 proteins of the co-culture system was higher than those of alone HCACC culture (*P = 0.001), but there was no difference on VEGF-A and VEGFR-2 expression between alone HCACC culture and the co-culture system. After treatment with NCTD, mF4-31C1 or NCTD + mF4-31C1, the expression of VEGF-C, VEGF-D and VEGFR-3 proteins of the co-culture system was downregulated significantly as compared to control group (all *P < 0.01), with lowest expression of these proteins in NCTD + mF4-31C1 group (^#^*P* < 0.01, *vs*. NCTD or mF4-31C1 group); whereas the expression of VEGF-A and VEGFR-2 proteins in NCTD or NCTD + mF4-31C1 group was lower than those of control or mF4-31C1 group (*P < 0.01), but there was no difference on VEGF-A and VEGFR-2 expression between control group and mF4-31C1 group, or between NCTD group and NCTD + mF4-31C1 group. **c** and **d** mRNA expression by RT-PCR: the expression of VEGF-C, VEGF-D and VEGFR-3 mRNAs of the co-culture system was higher than those of alone HCACC culture (*P = 0.001); but there was no difference on the expression VEGF-A and VEGFR-2 mRNAs between alone HCACC culture and the co-culture system. After treatment with NCTD, mF4-31C1 or NCTD + mF4-31C1, the expression of VEGF-C, VEGF-D and VEGFR-3 mRNAs of the co-culture system was downregulated significantly as compared to control group (all *P < 0.01), with lowest expression of these mRNAs in NCTD + mF4-31C1 group (^#^*P* < 0.01, *vs*. NCTD or mF4-31C1 group); whereas the expression of VEGF-A and VEGFR-2 mRNAs in NCTD or NCTD + mF4-31C1 group was lower than those of control or mF4-31C1 group (*P < 0.01), but there was no difference on the expression of VEGF-A and VEGFR-2 mRNAs between control group and mF4-31C1 group, or between NCTD group and NCTD + mF4-31C1 group

**Fig. 10:**
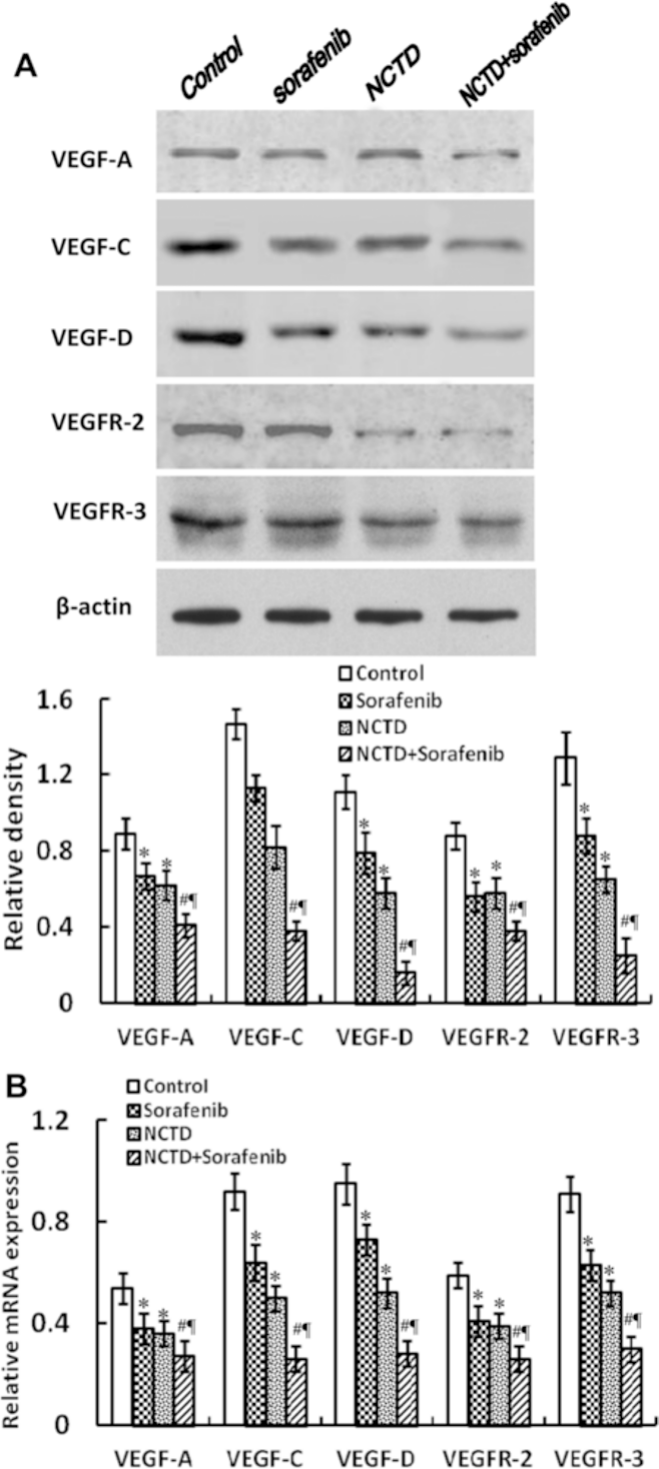
NCTD inhibits the expression of VEGF-A, VEGF-C, VEGF-D, VEGFR-2 and VEGFR-3 proteins/mRNAs of the *in-situ* colonic xenografts *in vivo.* **a** Western-blotting: the expression of VEGF-A, VEGF-C, VEGF-D, VEGFR-2 and VEGFR-3 proteins in NCTD, Sorafenib, or NCTD + Sorafenib group was significantly downregulated as compared to control group (*P < 0.01, ^#^*P* < 0.001), the expression of these proteins in NCTD + Sorafenib group was significantly lower than those of Sorafenib group or NCTD group (^**¶**^*P* < 0.05); but there was no difference on the expression of these proteins between NCTD group and Sorafenib group. **b** Fluorescent quantitative RT-PCR: the expression of VEGF-A, VEGF-C, VEGF-D, VEGFR-2 and VEGFR-3 mRNAs in NCTD, Sorafenib, or NCTD + Sorafenib group was significantly decreased as compared to control group (*P < 0.01, ^#^*P* < 0.001); whereas the expression of these mRNAs in NCTD + Sorafenib group was significantly lower than those of Sorafenib group or NCTD group (^**¶**^*P* < 0.05); but no difference on the expression of these mRNAs was observed between NCTD group and Sorafenib group

VEGF-C, VEGF-D and VEGFR-3, which were secreted from tumor and/or stromal cells, and the VEGF-C, −D/VEGFR-3 signaling pathways are believed to be the most important mechanisms underlying lymphatic endothelial cell growth and tumor lymphangiogennesis. VEGF-A, VEGFR-2, and the VEGF-A/VEGFR-2 signaling pathways are also reported to advance tumor lymphangiogenesis. To verify the anti-lymphangiogenic mechanisms of NCTD, we determined the expression of VEGF-A, VEGF-C, VEGF-D, VEGFR-2 and VEGFR-3 at protein and mRNA levels from HDLECs or the co-culture system *in vitro*, and the *in-situ* colonic xenografts *in vivo*. As shown in Fig. 8a and Fig. 9a and c, the expression of VEGF-C, VEGF-D and VEGFR-3 at protein product, protein and mRNA levels of the co-culture system *in vitro* was higher than those of alone HCACC culture (*P = 0.001), but there is no difference on the expression of VEGF-A and VEGFR-2 at protein product, protein and mRNA levels between alone HCACC culture and the co-culture system. After treatment, the expression of VEGF-C, VEGF-D and VEGFR-3 protein products, proteins or mRNAs was significantly downregulated in mF4-31C1, NCTD or NCTD + mF4-31C1 group as compared to control group (all *P* < 0.01), and the expression of these proteins or mRNAs in NCTD + mF4-31C1 group was lower than that of NCTD or mF4-31C1 group (all *P* < 0.01) (Fig. 8b and Fig. 9b and d); the expression of VEGF-A and VEGFR-2 protein products/proteins/mRNAs was also significantly downregulated in NCTD or NCTD + mF4-31C1 group as compared to control or mF4-31C1 group (*P* < 0.01), but there is no difference on VEGF-A and VEGFR-2 the expression between control group and mF4-31C1 group *in vitro* (all Fig. 8b and Fig. 9b and d). Furthermore, the expression of not only VEGF-C, VEGF-D and VEGFR-3 but also VEGF-A and VEGFR-2 at protein and mRNA levels of the *in-situ* colonic xenografts *in vivo* in NCTD, Sorafenib or NCTD + Sorafenib group was decreased significantly as compared to control group (all *P* < 0.01), with lowest expression of these proteins or mRNAs in NCTD + Sorafenib group (all *P* < 0.05) (Fig. 10), which was approximately in line with above expression of VEGF-A, VEGF-C, VEGF-D, VEGFR-2 and VEGFR-3 from the co-culture system *in vitro*. It was shown that HT-29 cells may promote VEGF-C, VEGF-D and VEGFR-3 secreted from tumor and/or stromal cells or indirectly promote VEGF-A and VEGFR-2 secreted from tumor and/or stromal cells, so accelerate the lymphatic tube formation of HDLECs, and the lymphangiogenesis and tumor growth of the *in-situ* colonic xenografts; NCTD or in combination with mF4-31C1 or Sorafenib markedly downregulated the expression of VEGF-C, VEGF-D and VEGFR-3 other than VEGF-A and VEGFR-2 proteins/mRNAs of the co-culture system *in vitro* and the *in-situ* colonic xenografts *in vivo*. Thus, we believe that NCTD inhibit tumor growth and lymphangiogenesis of HCACs *in vitro* and *in vivo* by downregulating the VEGF-C,-D/VEGFR-3 signaling pathway and the VEGF-A/VEGFR-2 signaling pathway.

## Discussion

As a small-molecule synthetic compound, NCTD has been reported to possess multiple potent antitumor properties in several cell lines, tumor xenograft models and human carcinomas; and is used selectively in clinic to treat many human malignant tumors by oral, intravenous injection or drip, or as a premedication or in combination with chemotherapy drugs for improving efficacy and reducing side effects in China because of its anticancer activities, fewer side effects and leukocytosis [3, 4, 9, 12, 15, 19, 23, 38, 51, 60–63]. However, these studies merely focused on effects of NCTD on the proliferation, apoptosis, growth and angiogenesis of a variety of human tumor cells *in vitro* and *in vivo*, including hepatoma HepG2 cells [55, 58], gallbladder cancer GBC-SD cells [8, 9, 12, 51, 61, 63], colon cancer HT29 cells [34], leukemia K562 [59] and HL-60 cells [21], and melanoma A375-S2 [1], and so on. In the experiment, on the base of our recent report in which NCTD suppressed lymphangiogenesis in human LECs [23], we further investigate the effect of NCTD on tumor lymphangiogenesis, and present evidence that NCTD inhibits tumor growth and lymphangiogenesis of HCACs by downregulating VEGF-C,-D/VEGFR-3 and VEGF-A/VEGFR-2 signaling pathways. This result is supported by observations listed below: 1) lymphatic tube formation from the 3-D culture of HDLECs and the co-culture system is observed *in vitro*; NCTD inhibits this lymphatic tube formation by suppressing proliferation, migration, invasion, tube formation of HDLECs in the co-culture system, inhibiting the expression of proliferation marker Ki-67 and anti-apoptotic gene Bcl-2, induced S-phase cell cycle arrest and cell apoptosis; 2) tumor lymphangiogenesis and lymphatic micrometastasis exist in the *in-situ* colonic xenografts *in vivo*; NCTD inhibits these tumor lymphangiogennesis and lymphatic metastasis; 3) VEGF-C, VEGF-D and VEGFR-3, and VEGF-A, VEGFR-2 proteins/mRNAs are highly expressed in the co-culture system *in vitro* and the *in-situ* colonic xenografts *in vivo*; whereas NCTD inhibits tumor growth and lymphangiogenesis by downregulating the expression of VEGF-C, VEGF-D and VEGFR-3, and VEGF-A, VEGFR-2 proteins/mRNAs. These results strongly support that NCTD serves as a potential antilymphangiogenic agent for tumor lymphangiogenesis of HCACs.

As the growth of newly lymphatic vessels in cancer, tumor lymphangiogenesis acts as a conduit by which disseminating tumor cells access regional lymph nodes, and lymph node tumor lymphangiogenesis and increased lymph flow through tumor-draining lymph nodes actively promote metastasis *via* the lymphatics [25, 31, 43]. It was reported that tumor lymphangiogenesis and the presence of metastatic cells in the sentinel lymph node are prognostic indicators of metastasis to lymph nodes, and the degree of dissemination determines the therapeutic course of action [64]. Thus, inhibition of tumor-associated lymphatic and lymphangiogenesis may be a key for controlling of primary tumor and metastasis [20, 25, 28, 31, 39, 43]. So, recent researchers have focused on hot-spots of antitumor by inhibiting tumor lymphangiogenesis [20, 28, 37, 64]. Some tumor lymphangiogenic inhibitors such as deguelin, endostar, silencing Id-1, liposomal honokiol, mF4-31C1 and sVEGFR3-Ig have been reported as adjuvant antilymphangiogenic and antitumor drugs against some metastatic cancers in experiment and in clinic setting [6, 7, 14, 16, 26, 35, 52]. But, there is still no experimental and clinical evidence whether NCTD can be used as a potential tumor lymphangiogenesis inhibitor. In this experiment, we further confirmed that NCTD not only has the antilymphangiogenic effect on human LECs, but also has the antilymphangiogenic activity against tumor growth and tumor lymphangiogenesis of HCACs *via* the 3-D culture of HDLECs and the co-culture system *in vitro*, and the *in-situ* colonic xenografts in nude mice *in vivo*.

Lymphatic tube formation i.e. lymphangiogenesis is a critical step for tumor lymphangiogenesis; whereas early stage of this lymphangiogenesis includes cell proliferation, apoptosis, migration and invasion of LECs [23, 25, 31, 43]. As a useful specific marker for detecting lymphangiogenesis or lymphatic micrometastasis and circulating carcinoma cells, LYVE-1 expressed on the surface of LECs plays an important role in the transportation of both lymphocytes and tumor cells into the lymphatic system, is considered to be the most valuable marker in identifying LECs and tissue lymphatics [17, 36]; Like LYVE-1, D2-40 (Podoplanin), which is an integrated cytoplasm/cell membrane mucoprotein mainly expressed in micro-LECs and lymphatic vessels and a relatively high specificity marker for LECs and lymphatic vessels, has now been used as value tools for the detection or prediction of tumor lymphangiogenesis and lymph node metastasis in human carcinomas [2, 13]; CK20, a type I cytoskeletal protein located on chromosome 17q21.2, have been known as a useful marker for detection of lymphatic micrometastasis and circulating carcinoma cells or for discrimination of the origin of metastatic tumors of unknown primary location such as micrometastasis in regional or sentinel lymph nodes and blood [29, 53, 54]. LMVD is the lymphatic microvessel density. Quantification of LMVD in the tumor might also be important for the evaluation of lymphangiogennesis, occult nodal metastasis (micrometastasis) and lymphatic metastasis in human carcinomas [40, 50]. We previously detected LYVE-1 expression, lymphatic microvessel and LMVD in human colorectal cancers, and evaluated their correlation with lymphangiogennesis, lymphatic metastasis, VEGF-C, −D and VEGFR-3, and prognosis in patients with colorectal cancer [10, 11, 22]. As a soluble VEGFR-3 antibody, mF4-31C1 has been shown the antilymphangiogenic activity for human LECs *in vitro* and the anti-tumor lymphangiogennesis *in vivo* [23, 36]. Sorafenib (Nexavar, a novel bi-aryl urea BAY 43–9006), is an oral multikinase inhibitor that blocks tumor cell proliferation and carcinogenesis by targeting the Raf/MEK/ERK signaling pathway and exerts an antiangiogenic effect by targeting several receptor tyrosine kinases including VEGFR-2, VEGFR-3 and platelet-derived growth factor receptor (PDGFR)-beta [37, 38]. Sorafenib has been demonstrated potent antitumor activities in studies *in vitro*, preclinical xenograft models of different tumor cells including human colonic adenocarcinoma cells *in vivo* and human clinical trials, has been clinically used for patients with advanced hepatocellular carcinoma, non-small-cell lung cancer, renal cancer and colonic cancer and demonstrated an improved overall survival in the patients [5, 44, 45]. Furthermore, Sorafenib has been used to enhance the antitumor effects and to overcome resistance by combination with chemoembolisation, chemoradiation and other antiangiogenic agents with different action mechanisms [30, 42, 56]. In view of the antilymphangiogenic activity of mF4-31C1 and the antitumor angiogenic or lymphangiogenic effects (targeting several receptor tyrosine kinases, including VEGFR-3) of Sorafenib, mF4-31C1 and Sorafenib were chosen as the experimental controls in this study. The results showed that NCTD, not only as mF4-31C1 *in vitro* [14, 23, 26, 35], inhibited the lymphatic tube formation of the 3-D culture of HDLECs and the co-culture system by suppressing proliferation, migration, invasion, and inducing apoptosis, as Sorafenib *in vivo* [37, 38, 62], inhibited tumor growth and lymphangiogennesis of *in-situ* colonic xenografts with a prolonged survival time (log-rank test, *P* = 0.026), but also downregulated the expression of LYVE-1, D2-40 and CK20 proteins/mRNAs, decreased the LMVD numbers in the *in-situ* colonic xenografts. Therefore, we believe that NCTD inhibits tumor growth and lymphangiogennesis of HCACs.

Tumors promote lymphangiogenesis by multifactor and complicated molecular mechanisms i.e., secreting molecules or cytokines such as lymphangiogenic growth factors from tumor and/or stromal cells that stimulate LEC growth and tumor lymphangiogennesis [20, 28, 37]. These lymphangiogenic growth factors and the regulators contributing to lymphangiogenesis mainly include VEGF-C, VEGF-D and VEGFR-3 [20, 25, 28, 37, 39]. Recently, VEGF-C, VEGF-D and their cognate receptor VEGFR-3 located on LECs and the VEGF-C, −D/VEGFR-3 signaling pathway are believed to be the most important lymphangiogenic growth factors and the most pivotal mechanism underlying tumor lymphangiogennesis [20, 25, 28, 37, 39]. VEGF-C or VEGF-D combines with VEGFR-3 located on LECs, thereby promoting proliferation of new lymphatic capillaries and tumor lymphangiogenesis *via* a series of intracellular signaling pathways. Clinical trials have indicated that the expression of VEGF-C, VEGF-D or VEGFR-3 is associated with lymph node metastasis and the poor prognosis of patients with cancer [10, 11, 22]. Relevant animal models have confirmed that VEGF-C, VEGF-D or VEGFR-3 plays an important role in the regulation of tumor lymphangiogenesis and lymph node metastasis. Activation of VEGF-C, −D/VEGFR-3 axis increases motility and invasiveness of LECs, promote formation of tumor lymphangiogenesis [20, 28, 37, 46]. So, some targeted strategies to block tumor lymphangiogenic pathways such as VEGF-C, −D/VEGFR-3 signaling pathway seem to be attractive anticancer treatment strategys. It was recently reported that Silencing Id-1 inhibits lymphangiogenesis through downregulation of VEGF-C in oral squamous cell carcinoma [6]; that liposomal honokiol and deguelin suppresses tumor lymphangiogenesis and lymphatic metastasis in xenograft tumor models by downregulation of VEGF-D both *in vitro* and *in vivo* [16, 52]; that blocking the expression of VEGFR-3 using interference vector-based RNA interference inhibits tumor growth of colorectal cancer [26]; that mF4-31C1 specifically inhibited both physiologically normal and tumor VEGF-C-enhanced lymphangiogenesis and new lymphatic growth in a 3-D culture of LECs and a mouse model of lymphatic regeneration by targeting VEGFR-3 [35]. Sorafenib blocked tumor proliferation and carcinogenesis by targeting inhibition of multi-signal pathways including VEGFR-3 [37, 38]. We lately reported NCTD, as mF4-31C1, inhibited the lymphangiogenesis of human LECs by simultaneously blocking VEGF-C, −D/VEGFR-3 pathways [23]. Actually, other than VEGF-C, VEGF-D and VEGFR-3, many other potent growth factors were involved in tumor lymphangiogenesis, such as VEGF-A, platelet-derived growth factor (PDGF), hepatocyte growth factor (HGF), angiopoietin (Ang)-1, Ang-2, insulin-like growth factor (IGF)-1 and basic fibroblast growth factor (bFGF), cyclooxygenase-2 (COX-2), and Slit2 [20, 24, 28, 32, 33, 37, 41, 46, 48, 57]. It is reported that Slit2, as a potent lymphangiogenic factor, contributes to tumor lymphatic metastasis [57]; VEGF-A treated lymphatic endothelial cell exhibited STAT3 activation in the nucleus, thereby enhancing lymphatic endothelial cell migration and increased tube formation (63). Indeed, many other growth factors such as VEGF-A, FGF-2, Ang-1, IGF-1 and HGF stimulate lymphangiogenesis indirectly through VEGF-C, VEGF-D or VEGFR-3 [20, 27, 28, 32, 33, 41, 48]. In the present experiments, we detected the expression of VEGF-A, VEGF-C, VEGF-D, VEGFR-2 and VEGFR-3 *via* immunohistochemistry staining, western blotting and RT-PCR. The results have shown that NCTD not only downregulated the expression of VEGF-C, VEGF-D and VEGFR-3 proteins/mRNAs but also inhibited the expression of VEGF-A and VEGFR-2 proteins/mRNAs of the co-culture system *in vitro* and the *in-situ* colonic xenografts *in vivo*; the downregulation of VEGF-C, −D/VEGFR-3 and VEGF-A/VEGFR-2 by NCTD in combination with mF4-31C1/Sorafenib can be enhanced. Thus, we believe that NCTD inhibit tumor growth and lymphangiogenesis of the co-culture system *in vitro* and the *in-situ* colonic xenografts *in vivo* by simultaneously blocking VEGF-C, VEGF-D and VEGFR-3 other than VEGF-A and VEGFR-2. Because there is a crosstalk between angiogenesis and lymphangiogenesis in tumor progression, we thus deduce that NCTD inhibits tumor growth and lymphangiogenesis of HCACs, as mF4-31C1 or Sorafenib through directly downregulating the VEGF-C, −D/VEGFR-3 signaling pathway, or similar to Sorafenib through indirectly downregulating the VEGF-A/VEGFR-2 signaling pathway. The present findings may be of importance to explore the therapeutically strategy of NCTD as an antilymphangiogenic agent for tumor lymphangiogenesis and lymphatic metastasis.

## Conclusions

Taken together, these results firstly show that NCTD inhibits the lymphatic tube formation of the co-culture system of HT-29 cells and HDLECs *in vitro*, tumor growth and lymphangiogenesis of the *in-situ* colonic xenografts *in vivo*; NCTD inhibits tumor growth and lymphangiogenesis of HCACs *via* multiple or “multi-points priming” mechanisms i.e. affecting proliferation, apoptosis, migration and invasion malignant phenotypes, inhibiting Ki-67 and Bcl-2 expression, inducing S-phase cell cycle arrest, and directly downregulating VEGF-C, −D/VEGFR-3 and/or indirectly downregulating VEGF-A/ VEGFR-2 signaling pathways. The results strongly suggest that NCTD may be a potential antilymphangiogenic agent for tumor lymphangiogenesis and can be explored for the prevention and treatment of tumor lymphatic metastasis of HCACs.

## Abbreviations

HCACs: Human colonic adenocarcinomas
LECs: Lymphatic endothelial cells
HDLECs: Human dermal lymphatic endothelial cells
NCTD: Norcantharidin
3-D culture: Three-dimensional culture
SABC: Streptomycin affinity biotin complex
S-P: Streparidin-peroxidase
RT-PCR: Reverse transcription- polymerase chain reaction
FCM: Flow cytometry
TEM: Transmission electron microscopy
LYVE-1: Lymphatic vessel endothelial hyaluronan receptor-1
CK20: Cytokeratin 20
LMVD: Lymphatic microvessel density
VEGF-A: VEGF-C, VEGF-D, VEGFR-2, VEGFR-3, vascular endothelial growth factor-A, −C, −D, −receptor 2, 3
